# Letter to the Editor

**DOI:** 10.1002/alz.70250

**Published:** 2025-05-12

**Authors:** Yan Li, Lon S. Schneider, Guoqiao Wang

**Affiliations:** ^1^ Department of Neurology School of Medicine Washington University St. Louis Missouri USA; ^2^ Department of Psychiatry and The Behavioral Sciences Department of Neurology Keck School of Medicine University of Southern California Los Angeles California USA

1

Dear Editor

In their paper, “Avoiding causal fraud in the evaluation of clinical benefits of treatments for Alzheimer's disease”,^1^ Dr. Liu et al. advocate that “the average between‐group difference in score change is where the debate and research efforts should be focused to contextualize and evaluate the clinical meaningfulness of the true treatment effect.” We agree with this recommendation but offer additional perspectives and clarifications to bolster it.

Liu et al. discuss various methods proposed to interpret between‐group differences in Alzheimer's disease (AD) trials. We previously reported that, when expressed as relative percentage reductions, these methods tend to converge on to similar numbers, reflecting a consistent underlying treatment effect despite differing presentations.^2^, ^3^ For instance, in the Clarity‐AD lecanemab trial, the between‐group difference of 0.45 points on the Clinical Dementia Rating Sum‐of Boxes (CDR®‐SB) corresponds to a 27% reduction *relative* to placebo decline (i.e., vertical, *y*‐axis comparison).^4^ The reported 5.3‐month “time savings” over the 18‐month trial is an algebraic transformation of the y‐axis value (difference in CDR‐SB) to the *x*‐axis (time) and equates to a 29% delay in progression of the CDR‐SB score relative to placebo (i.e., horizontal, *x*‐axis comparison), and the hazard ratio indicates a 31% reduction.^4^ These percentages—27%, 29%, and 31%—are closely aligned. This makes intuitive sense: analyzing the same data with various methods should not lead to dramatically different conclusions but instead should yield a similar *relative* percentage reduction, accounting for variations caused by the analytical approaches.

This pattern is also validated by Figure 1 in the stimulation study by Liu et al., where treatment (mean change = 1, SD = 0.7) and placebo (mean = 1.5, SD = 0.7) produce a 0.5‐point difference, or a 33% (0.5/1.5) *relative* reduction in progression. Using their 0.75‐point responder threshold, we recalculated and concluded that 64% of the treatment group versus 86% of the placebo group can be classified as “progressors,” yielding a 22% absolute risk reduction and a 26% relative risk reduction. At a 0.5‐point threshold (matching the mean difference), 76% of treatment versus 92% of placebo progressed, giving a 26% absolute and 28% relative reduction. These values 33% (mean difference), 26% (responder at 0.75), and 28% (responder at 0.5), are comparable, with the responder analysis at 0.75 producing the smallest percentage due to its conservative threshold, which captures a smaller part of the distribution. We previously reported that horizontal percentage reductions (i.e., *x*‐axis, time saved) could exceed vertical ones (i.e., *y*‐axis, CDR‐SB score) if placebo decline decelerates over fixed intervals (e.g., every 6 months) and is equal to or less than that of the treatment group.^5^ Thus far, however, this scenario has not been realized in AD trials unless the active treatment made patients perform worse than those on placebo. In other words, in recently reported, actual—not hypothetical or simulated^6^ —clinical trials, the use of “time savings” to express treatment effect does not increase or alter the nature of the treatment effect itself.

Liu et al. suggested that mixed models for repeated measures (MMRM) can account for within‐individual variation via random effects. Although incorporating random effects can be done,^7^ most MMRM analyses in recently reported AD clinical trials do not utilize random effects, focusing solely on between‐individual variation (e.g., baseline covariates and treatment groups).^4^, ^8^, ^9^ On the other hand, responder analyses could similarly incorporate covariates and random effects for longitudinal binary outcomes to control for both between‐individual variation and within‐individual variation. Therefore, statistically, MMRM is not inherently superior to responder approaches, and its strength lies in preserving continuous data, not dichotomizing continuous data.

In summary, whether using mean differences, time savings, or responder thresholds, relative percentage reductions converge on a similar magnitude, reflecting the same underlying *absolute* treatment effect.^2^ The average between‐group difference remains the criterion or “gold standard” from which efficacy inferences are made in randomized clinical trials, as Liu et al. argue, and should be the focus to contextualize the clinical meaningfulness of the true treatment effect. To restate the obvious, the treatment effects on the CDR‐SB in the lecanemab Clarity‐AD phase 3 trial is 0.45 CDR‐SB points, and in the donanemab TRAILBLAZER‐ALZ‐2 phase 3 trial is 0.67 CDR‐SB points (low–medium tau population).^4^, ^9^ Algebraic transformations of these outcomes could sometimes facilitate interpretation, but they neither amplify nor alter the fundamental treatment effect.

Finally, clinical trials that would aim to determine clinically meaningful change^10^ or individual patient benefit should consider a time to event or progression free survival design whereby any change on the outcome can be accepted by most experts as clinically meaningful.

## CONFLICT OF INTEREST STATEMENT

Guoqiao Wang, PhD, reports currently serving on a Data Safety Committee for Amydis Corporate, Abata Therapeutics, and statistical consultant for Eisai inc. and Alector Inc. All the other authors reported no conflicts of interest. Author disclosures are available in the Supporting Information.

## FUNDING INFORMATION

Lon Schneider, M.D., reports within a 36 month time frame, grants from NIH R01 AG054434 (Delivery of Essential Fatty Acids to the Brain in Alzheimer's disease), P30 AG066530 (USC ADRC), R01 AG062687, R01 AG051346, R01 AG055444, P01 AG02350, R01 AG053267 (DIAN‐TU), R01 AG074983 (Aβ and tau vaccines), R01 AG063826, State of California CADC 15‐10291; grants and personal fees from Eli Lilly/Avid, grants and personal fees from Roche/Genentech; grants/contracts from Eisai, Biogen, and Biohaven; grants from Washington University, St. Louis/ NIA DIAN‐TU, grants from UC San Diego ADCS, personal fees from Neurim, Ltd (Israel), Cognition Therapeutics, Corium, Immunobrain Checkpoint, Ltd (Israel), Alpha‐cognition, BioVie, Lexeo, Lighthouse, AC Immune (Suisse), Athira, GW Research (UK, Jazz, USA), Merck, Otsuka (USA), Pharmatrophix, Linus Health, Lundbeck, Novo Nordisk, Muna (Denmark), Ono Ltd (Japan), Vivli.org, outside the submitted work; and from The Della Martin Foundation endowment. Guoqiao Wang reports receiving grant support from NIH: U01AG042791, U01AG042791‐S1 (FNIH and Accelerating Medicines Partnership), R01AG046179, R01AG053267, R01AG053267‐S1, R01AG068319, and 1U01AG059798.

## Supporting information



Supporting Information
